# Patient-centred care: reality or rhetoric—patients’ experiences at ARV clinics located in public hospitals in KwaZulu-Natal, South Africa

**DOI:** 10.1186/s12981-022-00463-2

**Published:** 2022-09-10

**Authors:** Delarise M. Mulqueeny, Myra Taylor

**Affiliations:** 1 Department of Social Work, Faculty of Arts, University of Zululand, Private Bag X1001, Room 107, Faculty of Arts Building, 1 Main Road, Vulindlela, KwaDlangezwa, 3886 Zululand South Africa; 2 Discipline of Public Health Medicine, School of Nursing and Public Health, Howard College, University of KwaZulu-Natal, Durban, 4001 South Africa

**Keywords:** Patient-centred care, ART, Patients experiences, KwaZulu-Natal, Public hospitals

## Abstract

**Background:**

The South African public antiretroviral therapy (ART) programme is considered one of the largest and most successful ART programmes worldwide. Hence, a study exploring the patients’ experiences of the public antiretroviral therapy (ART) programme in the second decade of the programme is relevant as no study has been published on patients’ experiences at these sites.

**Objectives:**

To explore patients’ experiences of care in the public ART programme at four ARV clinics within the eThekwini District, KwaZulu-Natal.

**Method:**

A mixed-methods study design with 12 in-depth patient interviews, non-participatory observation, and a stratified random sample of 400 patients completed questionnaires. Qualitative data were thematically analysed. Quantitative data were analysed using a SPSS 24 package to determine frequencies and differences in patients’ responses (p < 0.05). The socio-ecological model framed the study.

**Results:**

All 412 patients reported valuing the provision of free ARVs. Patients’ positive experiences included: routine blood results mostly being available, most staff greeted patients, there were sufficient nurses, patients were satisfied with the time that they spent with doctors, clean clinics, and private and safe counselling areas. The negative experiences included: poor relationships with nurses, negative staff attitudes, disrespectful staff, information was lacking, inadequate counselling at times, varying and inflexible appointments, challenges with data capture and registration systems; varying ARV collection frequencies, routine health tests and processes per site, and the absence of patient committees and representatives.

**Conclusion:**

The results reflected positive and negative experiences which varied between the facilities, as processes and systems differed at each site. Innovative patient-centred processes and programmes could be implemented to ensure patients have mostly positive experiences. As part of continuous improvement, patients’ experiences should be regularly explored to ensure that the ART programme meets their needs and expectations.

## Introduction

South Africa has one of the world’s largest HIV populations with an estimated prevalence of 14.0% and 7.8 million people living with HIV (PLHIV) [1]. In South Africa most people, including PLHIV, rely on the public health care system for access to treatment [2, 3]. The public antiretroviral therapy (ART) programme was established in 2004 and provides parallel services in their general health facilities. The programme was scaled up in 2016 to include all PLHIV, regardless of their CD4 count or clinical stage [4, 5]. The increased availability of ART resulted in decreased rates of opportunistic infections and HIV-related mortality and morbidity and enabled PLHIV to live healthier, longer lives, establish families, be gainfully employed and to a great extent live normal lives [6, 7].

However, despite the country achieving great success in reducing HIV-related mortality and morbidity, the scale up and high HIV prevalence further strained an already overburdened health care system and challenged existing resources [4, 8]. Such challenges include stockouts, poor infrastructure and systems and insufficient and inefficient staff to name a few [9]. Worth noting, PLHIV require comprehensive HIV care. Hence, quality support and care, inclusive of the management of adherence, co-morbidities, opportunistic infections, nutritional deficiencies, antiretroviral (ARV) side-effects, the provision of palliative care, as well as assistance with spiritual, financial, and psychosocial challenges are required [10, 11]. Although patients are an important part of the ART programme which treats a chronic disease that requires a lifetime of daily treatment, many studies predominantly report on patients’ outcomes. These include ART adherence, loss to follow-up and stigma with PLHIV. However, assessing patients’ outcomes and experiences of the ART programme have frequently been excluded [12, 13]. An example of the benefits of reviewing patients’ experiences of care is a study conducted by Grocott and McSherry that explored how the involvement of patients can inform clinical changes [14]. The advantages of such an approach are also noted in Doyle’s systematic review of studies that focussed on patients’ perspectives to emphasize the benefits of improving safety and clinical effectiveness [15].

Hence, this study is relevant and differs by providing a patient-centred perspective of patients’ experiences that includes their challenges and successes in a mature ART programme. Thereby, providing a balanced perspective, which is important to identify and understand the changes that could be implemented to improve the facility, systems, care, and service delivery. This study has relevance for regional and local audiences as it embraces and values the end-users’ experiences and highlights the importance of patient-centred care (PCC). 

Patients’ experiences of the public ART programme at the four ARV clinics in public hospitals in the eThekwini district, KwaZulu-Natal (KZN), South Africa include: (1) factors associated with self-reported ART adherence, (2) disclosure of HIV status, (3) interpersonal factors about their observations and concerns regarding HIV-knowledge dissemination, (4) relationships and communication with clinic staff and (5) factors affecting service delivery. The research question addressed was: what are PLHIV experiences at ARV clinics at public hospitals?

## Methods

### Study design

The study utilised an explanatory sequential mixed-methods design with the quantitative component comprising of closed-ended questionnaires, that preceded the qualitative in-depth open-ended interviews. This was done to ensure that potential information gaps were comprehensively explored, and comprehensively characterised patients’ experiences. An adapted socio-ecological conceptual framework was utilised as various socio-ecological factors (individual, interpersonal, institutional, policy) impact on patients’ experiences.

### Study setting

The study setting was four (three regional and one district) public hospitals in eThekwini District, KZN that serves different geographical areas within the health district. Doctors, administrators, nurses, HIV lay counsellors, pharmacists all form part of the clinics. In some clinics, social workers and pharmacists provide services to the patients but they are not based inside the clinic but within the hospital grounds. At each ART clinic, patients started off by registering and collecting their receipts and files from the hospital’s administration section. Thereafter they joined the ART clinic queue to see a doctor. Nurses provided support to the attending doctors and facilitate the queuing procedures for patients waiting to see doctors. After their consultation, patients proceeded to the pharmacy to collect their medication. Where necessary, separate appointments had to be made to see social workers. Each hospital had its own format and process regarding how the above was achieved.

### Study procedures and eligibility criteria

The eligibility criteria were that participants had to be HIV-positive patients, 18 years and older who had accessed ART at the study sites for a year and more as ART-literacy was a requirement for all participants. This was to provide comprehensive data regarding their experiences. The sample size included 400 patients (100 per site using stratified random sampling of every fourth patient at each ARV clinic) completing the questionnaires and purposive sampling of 12 patients (three patients from each site) who participated in the in-depth interviews (IDIs). The questionnaire was piloted with patients and revised using a participatory action research approach (PAR) with the principal investigator (PI), the co-investigators. This resulted in four patients developing a new questionnaire.

Patients at each of these hospitals reported on institutional ART procedures which included the infrastructure, facility, administration systems and processes, clinic attendance frequency, stock outs, service integration and patient representation.

### Ethical considerations

The data collection process only commenced after ethical and site clearance was received from the relevant University’s ethics committee, the Department of Health and all four hospital managers.

### Data collection and management

Data collection occurred over a 3-month period, in 2015. Confidentiality and anonymity were explained and written informed consent was sought and received from all 412 respondents. The researcher and two trilingual research assistants (Afrikaans, isiZulu, and English) administered the 400 questionnaires. Thereafter, the researcher conducted the 12 dual-recorded in-depth interviews (IDIs) utilising an interview guide. The assistants were present to address any cultural and language barriers and to ensure reliable data collection.

## Data analysis

Each questionnaire was checked for completeness to ensure accuracy. The quantitative data was captured in the Epidata software programme, and the Statistical Package for the Social Science (SPSS 24) programme was used to analyse the data. Descriptive analyses are reported as frequencies and percentages, and bivariate analyses used chi square for categorical data and analysis of variance for continuous variables for the parametric data. For nonparametric data, the Kruskal Wallis test was used to compare the results from the four hospitals. The level of statistical significance was p < 0.05.

The IDIs were transcribed, coded, and analysed utilising Creswell’s approach to ensure reliability [16]. Patients’ responses were assigned codes according to their sex [female (F) and male (M)] and the four hospitals (hospital 1, 2, 3 and 4). Themes from the socio-ecological framework (individual, interpersonal, institutional and policy) allowed for an integrated presentation of the findings. The co-author verified the transcription of the interviews and the thematic analysis. Purposive sampling of three patients from each site for the in-depth interviews assisted with transferability, by comparing different sources (questionnaires and IDIs) of the data [17]. These were then included in the triangulation of the data.

## Results

The patients’ socio-demographic characteristics are contained in Table 1. Most patients were between the ages of 35–44 years and ages differed slightly between hospitals (p < 0.005). From the quantitative results, more women required their appointments to be made telephonically (p < 0.005).

**Table 1:** Socio-demographic details of patients who responded to the questionnaire by hospital n = 400

Option	Hospital 1	Hospital 2	Hospital 3	Hospital 4	n = 400	%
Age*						
18–25	10	7	7	5	29	7.3
26–34	24	27	23	20	94	23.5
35–44	39	43	36	29	147	36.8
45–59	19	19	29	45	112	28.0
60–64	5	2	5	1	13	3.2
65 and older	3	2	0	0	5	1.2
Ethnicity*						
Black	82	97	58	63	300	75.0
Indian	6	0	35	7	48	12.0
Coloured	8	3	6	25	42	10.5
White	2	0	1	4	7	1.8
Other	2	0	0	1	3	0.8
Sex						
Female	60	54	69	56	239	59.8
Male	40	46	31	44	161	40.2
Sexual orientation						
Heterosexual	92	95	99	94	380	95.0
Same-sex partnership	7	5	1	6	19	4.8
Transgender	1	0	0	0	1	0.2
Educational level**						
Grade 7 and lower	15	6	22	19	62	15.5
Grade 8–12	30	38	40	44	152	38.0
Completed grade 12	40	43	36	31	150	37.5
Diploma or degree	15	13	2	6	36	9.0

*p < 0.005, **p = 0.002

The IDI patients comprised of six males and females and three patients from each of the four hospitals.

## Patients’ individual experiences

### ART adherence

The patients had accessed ART from their clinic for a mean of 3.6 years (standard deviation, (SD) 1.6). Over half the participants (56.5%) had taken their ARV medication since first initiating treatment, which on average was 3.6 years ago. The original reported ART adherence was significantly (65%) better at hospital 3, than at the other hospitals (52.0% at hospital 1, 53.0% at hospital 2, and 56.0% at hospital 4) (p = 0.046). Medication adherence had improved to 83.8% at data collection, with no significant differences between the four clinics.

### Disclosure of HIV status

Most patients only disclosed their HIV status to people they felt comfortable with, to keep their HIV status confidential. This ranged from 47.0% respondents at hospital 2 to–75.0% at hospital 4. Non-disclosure was associated with attending ARV clinics where patients collected their main patient file from the main hospital (p = 0.046), and these patients preferred a separate ARV clinic for privacy (p = 0.003). There was no association between patients’ disclosure and reported ART adherence.

The study sample for the interviews incidentally included patients of varied sexual orientation, ages, racial groups, and gender representation.

### Experiences with doctors and dieticians

Although 51% of patients reported seeing a doctor every three months due to side effects or medication changes, 42.5% respondents were informed about the potential side-effects. However, this differed significantly by institution (p = 0.033). (Table 2).

**Table 2:** Patients’ experiences with the services provided by the doctors/dieticians and counsellors n = 400

Option	Hospital 1	Hospital 2	Hospital 3	Hospital 4	n = 400	%
Doctor/dieticians						
Are patients allowed to ask doctors questions and provide input regarding their illness/treatment?*						
Yes	20	46	28	12	106	26.5
No	3	1	0	0	4	1.0
Some do	**77**	53	72	88	290	72.5
How many times have doctors/dieticians discussed patients’ diet at the ARV clinics?**						
Once	35	26	31	25	117	29.3
Twice	18	11	6	6	41	10.3
Three times	1	2	0	4	7	1.8
Never	46	61	63	65	235	58.8
How often do patients see a doctor?*						
Every month	0	0	0	3	3	0.8
Twice a year	100	54	26	10	190	47.5
Quarterly	0	46	72	87	205	51.3
On request to see one	0	0	2	0	2	0.5
Are there sufficient doctors at their ARV clinics?*						
Yes	42	25	96	0	163	40.8
No	0	7	1	77	85	21.3
Sometimes	58	68	3	23	152	38.0
Do doctors explain to patients why their medication is being changed?						
Yes	54	60	70	68	252	63.0
No	1	0	0	1	2	0.5
Medication unchanged	45	40	30	31	146	36.5
Are medication side effects explained to patients***						
Yes	31	43	50	46	170	42.5
No	24	0	20	23	84	21.0
Medication unchanged	45	40	30	31	146	36.5
Do patients trust the counsellors to keep their information confidential?*						
Yes	67	64	85	83	299	74.8
Do counsellors know enough about HIV and ART to counsel patients?*						
Yes	40	54	38	26	158	39.5
No	10	8	1	4	23	5.8
Some do	50	38	61	70	219	54.8
Do counsellors discuss dating option and potential dating challenges?						
No	100	100	100	100	400	100
Are patients comfortable to discuss sex and reproductive health with counsellors?						
Yes	60	73	79	69	281	70.3

*p < 0.005, **p < 0.05, ***p = 0.033

Doctors spent between 10 and 15 minutes with 53.3% of the patients and 97.5% were satisfied with this time. Most (99.0%) patients were allowed to see a doctor when it was not their appointment day with 56.8% reporting not seeing the same doctor on each clinic visit. Whilst 35.8% stated that doctors greeted them when they entered their consulting rooms, 26.5% reported that doctors allowed them to ask questions or give input regarding their illness. In the IDIs there were different views.

**IDIs:** *No, they don’t, as they just want to get rid of us quickly. Most of them barely greet us so they would never waste their time asking us for our opinions. They don’t even look at us in our eyes, so we are not comfortable asking them questions. (M1)*

*On most days there are not enough doctors. (M4)*

Only 19.3% of the patients were physically examined by a doctor since ART initiation. Most patients equated a doctor’s examination with better care and expressed disappointment that doctors carried stethoscopes, yet most did not examine patients. One patient questioned the need for doctors to see patients as he felt administrators or nurses could respond similarly.

**IDIs:** *The doctors carry stethoscopes, yet they never examine us. I have never been examined since I came here. (F1)*

*Doctors normally examine patients, but these only look at our files. They should check the files and if there is a problem speak to us as admin people or nurses would be able to ask us the same two questions that they ask us: Are you using condoms? Do you take your tablets on time? (M4)*

### Experiences with counsellors

Over 62% of the respondents were counselled for between 30 and 60 min with significant statistically differences in patients’ responses regarding confidentiality (where hospitals 1 and 2 had lower scores) (p < 0.005). Patients at the hospitals had significantly different levels of confidence in counsellors’ knowledge of HIV and AIDS (p < 0.05). The IDIs revealed that some participants reported poor provider-patient relationships and discomfort with counsellors’ ages.

**IDIs***: The counselling is not good—just that we don’t know what is expected of counsellors. (M4)*

*I can’t tell someone who is my child’s age, personal stuff. Young people like them have not lived long enough and they certainly don’t have enough experience to counsel us oldies. (M2)*

Whilst the questionnaire respondents reported that challenges in intimate partner relationships were not discussed with them, the IDIs show that dating and sexual relationships were an integral part of patients’ lives. The interviewees required trained staff to counsel them about navigating these relationships and on how to handle rejection and stigma.

**IDIs:** *I want to be advised about dating as I have sexual needs which need to be met. Sex does not end because I am HIV-positive. I need to know what to say without a man disclosing my status to others or rejecting me. (F3)*

Only Hospital 4 patients had their ARV pill count conducted by counsellors and an interviewed respondent viewed this as a waste of time, as experienced patients could discard their unused ARVs.

### Experiences with nurses

Across all hospitals, 97% of the patients indicated that there were adequate numbers of nurses. Hospital 3 patients accounted for 40% of the patients who observed health talks whilst some hospital 2 and 4 patients and one patient from hospital 1 had observed health talks since the programme first commenced and on special days like World Tuberculosis Day and World AIDS Day. Less than half the respondents (40.0%) were confident that the nurses had sufficient HIV knowledge to treat them as compared to that of counsellors (39.5%).

### Experiences with social workers

Most patients, 77.8% had not interacted with a social worker since attending their ARV clinic.

This could be attributed to Hospital 3 having a social worker permanently stationed at the ARV clinic, whilst Hospital 4’s ARV social worker was stationed at the Social Work department and attended the ARV clinic at specific times daily. Hospital 1 and 2’s patients accessed the social work departments for services. Two patients expressed challenges to see social workers, as appointments were required to see one, which meant an additional hospital visit with financial implications. Only the IDIs revealed that patients experienced family and economic issues.

**IDIs**: *Things are not easy at home. My child is on drugs and my husband doesn’t care. We have problems and it’s not easy to see a social worker. (F1)*

*The social workers are separate from the clinic, so we must make appointments and return again. (M3)*

## Patient satisfaction with administrative, clinical, and counselling staff

Of hospital 1’s patients, 85.0% reported challenges accessing staff to answer their questions and to provide assistance and 50% observed staff using their cell phones, which was more frequently reported than at other hospitals. Similarly, 81.8% of the patients expressed dissatisfaction when staff used their cell phones during consultations, with the highest (88.0%) recorded at hospital 4. Two interviewed patients recommended more vigilant supervision and cameras be installed to monitor occurrences at the clinic. The IDIs reflect a satisfied Hospital 3 patient which contrasted with patients at other hospitals:

**IDIs:** *Our staff are great. I cannot complain. They take the time to know us and our stories. (F3)*

*If we go and ask staff why we are waiting so long, most are rude. (M1)*

Although 25.3% patients had experienced rude or unhelpful staff members, only 2% had reported such behaviour as they feared reporting staff due to them being discriminated against.

**IDIs:** *It is hard to report them because we must come back here again. They might keep our files back or say our files are lost. So, our situation could get worse for reporting them. (M1)*

*I’m scared to report them as they will ill-treat us more. Also reporting them is not going to make things better. At the end of the day staff won’t get fired for not doing their jobs or being rude. (M4)*

## Experiences of the hospital’s ART programme

Some patients were comfortable receiving treatment in separate ARV clinics as their HIV status connected them to other patients. Hence, they were free to discuss their status, treatment, and lives without feeling stigmatised whilst waiting to be attended to.

**IDIs:** *I’m happy that we have a separate clinic as we all have HIV and can talk to each other about things that we are going through…We all in the same boat so no one is looking at the next person in a funny way. (F2)*

The current paper-based system was perceived as outdated and electronic and computerised systems were required to speed up processes.

**IDI:** *Today most systems are computerised. Here we must wait for receipts, wait for files and nurses and doctors write in our files. The programme is electronically outdated. (M1)*

*To save time, all our information should be computerised. (F1)*

### ARV collection frequency

Eighty percent of all patients collected their ARVs monthly, with 76.8% expressing dissatisfaction about doing so. The economic consequences of frequent clinic visits were highlighted, another felt that stable patients should receive ARVs for 2–3 months. Another patient was aware of the Central Chronic Medication Dispensing and Distribution (CCMDD) programme, a new and novel initiative, but was unsure how and when it would be rolled out.

**IDI**: *We appreciate free ARVs but its costly coming here every month. (M2)*

ARV stock outs during the data collection process contributed to some hospital 3 and 4 patients collecting ARVs monthly instead of every two to three months.

**IDIs:** *They’ve been out of Abacavir for too long now, so I come here monthly which costs money. (F3)*

The pharmacy’s location whether a part of the clinic, separate from the ARV clinic or a separate window in the main hospital were a concern as patients were sensitive to being on ART and felt their HIV status was being exposed by these structural differences. Two interviewed patients differed regarding ARV collection procedures, as one preferred collecting from an ARV pharmacy within the ARV clinic whilst the other preferred collecting hers from an integrated pharmacy to avoid people knowing her status.

**IDIs:** *I really would like to see a doctor and collect my ARVs in one place. (M1)*

*It’s easy for people to know we are HIV-positive because we receive our ARVs separately from others. (F1)*

At hospitals 2 and 4, 75% of patients collected their ARVs in less than half an hour, whereas only 27% of hospital 3 and 47% of hospital 1’s patients received their medication in this time. The remaining patients at all the hospitals waited up to an hour with these differences being statistically significant (p < 0.005).

All patients reported clean clinics, considered counselling facilities safe and confidential while hospitals 2, 3 and 4 reported that their waiting areas were unprotected from the elements. Additionally, there were often delays in patients obtaining their medical files. Patients indicated different procedures for file collections and returns at each hospital, which they considered time-consuming. A fifth of the patients reported lost or misplaced files, which was also emphasized in the IDIs:

**IDIs:** *I cannot tell you how many times they have lost my file. We … go down to the ground floor and stand in another queue. That’s takes a long time. (M1)*

*This back and forth filing system is crazy. (M2)*

### Patients’ representation

Patients did not feel that the system and staff had their interests, uniqueness, and adherence at the core of service delivery. They highlighted that patients should play advocacy roles or be recruited as ‘patient representatives’ as they had several years of experience accessing the ARV clinics and being on ART. They further reported that the non-existence of patient fora reflected that patients are not at the epicentre of their care and treatment as their concerns were not sourced nor perceived as important.

**IDIs:** *There are no patient forums which shows that our feelings and thoughts are not important here. (M3)*

## Patients’ experiences

Most interviewed patients reported negative experiences and such experiences mostly related to staff than systems, policies, and processes.

## Discussion

Patients’ experiences seen from their perspective are critical indicators of patient satisfaction, patient-centred care, and quality of care and hence useful for improvement and change strategies [12, 18]. The study results, emphasise that although patients appreciate the provision of the free lifesaving ARV drugs, they are dissatisfied with the service provision. The socio-ecological model was adapted to include only four categories [19]. The first category (individual) addressed patients’ knowledge, attitudes, observations, educational and financial levels, literacy and reports on their adherence and HIV status disclosure. The second category (interpersonal) refers to their identity as HIV-positive patients and their interactions and relationships with health care providers, patients’ peers, and communication at the ARV clinics. The third (institutional) pertains to the hospital and clinic systems, processes, and rules and the fourth relates to the national and local policies of the ART programme and the clinics.

The availability of free ART to South African PLHIV has resulted in HIV being a manageable chronic illness and improved patients’ quality of life. However, free ART is insufficient if patients are not adhering on ART due to negative experiences as this could result in multi-drug resistance occurring which impacts on patients and the health care system [20]. An adherence rate of ≥ 95% is often cited as a requirement for virological success [21]. However, the study results reflect an adherence rate of 83.8% during 2015 across the four sites. The patients’ previous non-adherence could be a reason for their complications, more frequent accessing of facilities and them still accessing treatment from a hospital ARV clinic rather than at a community clinic. Socio-demographic, cultural, economic factors and inadequate health systems are all factors that impact on ART adherence [22]. Adherence strategies cited in other studies are adherence clubs, pillboxes, stringent adherence counselling by all health workers, alarms, mass media campaigns, utilising ‘expert patients’ and peer supporters [23–26].

### Disclosure of HIV-positive status

Although disclosure is a key element in the management of HIV, it is primarily an individual’s decision based on and influenced by multifactorial factors. This study’s results concur and show that most patients disclose their status to people they felt comfortable with to maintain the confidentiality of their status [27]. However, separate ARV pharmacies and non-integrated systems at the clinics compelled patients’ status to be disclosed. This has been similarly documented in a Western Cape qualitative study that addressed ART adherence barriers and found that many patients defaulted because their status “could unintentionally be disclosed if they were seen in queues at the ARV clinics regularly” [28]. Similarly, a study conducted at a South Indian tertiary-care hospital found that non-disclosing patients’ travelled long distances to access facilities far from their homes in endeavours to hide their HIV-positive status which has financial implications [29].

### Gender

More South African women than men are on ART and studies show that women reflect better virological responses to ART than men due to the latter accessing treatment later [30, 31]. This study’s quantitative results concur with more women accessing the ARV clinics and being on treatment but there were no significant differences between the four ARV clinics in this study.

## Patients’ interpersonal experiences

Besides patients, staff constitute a major component of the ART programme. Hence, the importance of patients’ experiences and perceptions being articulated. Respondents’ interpersonal experiences include verbal and non-verbal interactions with nurses, clerks, doctors, social workers, lay counsellors, pharmacists, and pharmacy assistants. Patients’ responses highlight that they access a mostly paternal health system where health professionals have more power than patients and staff’s behaviour and attitudes can positively or negatively impact on them. Several South African studies document similar interpersonal relationships [32–34].

### Experiences with doctors and dieticians

The qualitative responses concur with the quantitative data regarding patients reporting that most doctors do not allow them to ask questions or source their input regarding their illness. Most hospital 4 respondents reported on the shortage of doctors which concurs with reports on the shortage of South African public health doctors [35]. Dieticians assist patients to obtain optimal nutritional intake by imparting relevant dietary and nutritional information to them [36]. However, this study’s results highlight a gap as more than half the patients reported not discussing their diet with a health care professional. This could be attributed to patients having forgotten discussing their diet, having a positive body mass index (BMI), the substantive distance between the dietician department and the ARV clinics and patients having to make extra appointments with financial and time implications.

### Experiences with counsellors

HIV education and counselling are important components of the ART programme as they aid in patient empowerment, education, and adherence [37]. However, some patients highlighted information gaps (dating) which can be linked to a study that found counsellors’ non- adherence to a patient-centred counselling approach nor to Egan’s model of counselling. The strengths of using either approach is the counsellor-patient partnership that empowers patients to manage their HIV diagnosis and treatment adherence by understanding their needs [38]. Both these approaches place patients at the epicentre of their treatment with counsellors assisting them to effectively manage their ART journey. Motivational Interviewing also improves behaviour change and it could be used to probe for more information and improve patient experiences, adherence, and health management. It could also be implemented in all health care workers’ counselling, information sharing and support sessions [39].

### Experiences with nurses

A respondent’s report that nurses engaged in administrative, and computer work rather than nursing duties has been documented in another study [40]. Most respondents expressed dissatisfaction with staff members using personal communication devices whilst dealing with them and concurs with another study’s finding that mobile phones can be a distraction, breach confidentiality and affect hygiene, efficiency and patient experiences and care. That study, recommended monitoring the use of personal instruments as a prevention strategy [41]. However, other studies have highlighted benefits to HCWs using communication devices to access information at point-of-care [42, 43]. The reported benefits of health talks are like that of a study conducted at a Johannesburg HIV/AIDS clinic which stressed that HIV management requires quality, relevant information to ensure patients are well informed to live meaningful lives [44].

## Experiences with administrative, clinical, and counselling staff

From the results, health service providers and patients’ communication posed a challenge as a quarter of the patients had encountered rude and unhelpful staff. Few patients reported such behaviour due to their regular facility visits, fear of intimidation and further ill-treatment. This concurs with the findings of other studies [45, 46]. Whilst patients from other studies cited staff’s unprofessional behaviour as a reason for their non-return to the clinics, this study did not address loss to follow-up patients and respondents did not report such. The results reflect the importance of characteristics such as empathy, caring and approachability to patients [47]. They also highlight the almost non-existence of positive relationships, which reflects a gap in care, as relationship building between staff and patients is important for patient retention, adherence and quality care [12] The mostly positive experiences of hospital 3 patients could be attributed to effective leadership and communication and the clinic manager’s participatory leadership style [48].

To summarise, positive interpersonal experiences within the public sector, is attainable. However, it requires commitment from health workers, a strong internal locus of control, training, dialogue between patient and providers, corrective or disciplinary action for non-performing staff members and continuous monitoring, supervision, and assessments to ensure strengthened and satisfactory interpersonal relationships.

## Patients’ institutional experiences of the hospitals’ ART programme

### Infrastructure, facility, systems, and processes

The provision of ART was initiated through a vertical programme and some interviewed patients expressed the need for a more integrated service. However, these patients were in the minority. Although patients were screened for TB, TB treatment and the challenges of treating PLHIV with TB was not reported even though KZN has high rates of comorbidities which could further burden the public ART programme [49]. Hence, the need for vigilant effective health providers to monitor the situation.

The results emphasized service challenges including patients making telephonic appointments, the whereabouts/location of the ARV pharmacies and medication collection processes and the lack of available seating for waiting patients in the mornings. Patients still reported inadequate infrastructure years into democracy and over a decade after the ART programme rollout, despite the impact that a negative environment could have on patients’ psyche [50]. Worth noting is that all patients found the facilities to be clean and felt safe within the counselling workstations. This is similar to the findings of a South African study conducted in Vhembe District [12]. Interestingly, pill counts are an indication of adherence, yet this is only practised at one of the sites [51]. Despite stock outs, few respondents reported receiving short supplies of ARVs in this study [52]. The Chronic Medicine Dispensing and Distribution (CCMDD) programme had not been implemented during the data collection process [53]. This intervention could improve patients’ ARV collection experiences as they would be able to access treatment from alternate distribution centres at times and locations suitable to them. Economic challenges could also be appeased by this programme.

A shortage of permanent doctors was mostly reported at hospital 4. However, this shortage has also been recorded in other South African public hospitals and clinics [54]. Nurse-initiated management of antiretroviral therapy (NIMART) has been successfully highlighted in other studies [55, 56]. Although NIMART is not implemented at hospital ARV clinics, its rollout could address doctors’ shortages.

### ART collection frequency

Although ARVs are free at all four hospitals, patients attend ARV clinics and pharmacies between two and twelve times per annum and these visits have cost implications, which challenge patients’ adherence, financial budgets, and livelihood. This concurs with a study that measured the cost of obtaining free ART in the South African public sector [57]. Additionally, studies have highlighted transportation costs as a barrier to ARV uptake and adherence [58, 59].

### Patient representation

Putting patients at the forefront of their health care journey requires the inclusion of patients’ perspectives on every aspect of their care, treatment and support as their input articulates their unique needs and experiences [60]. However, patients reported that they felt excluded, their input was not sourced, their uniqueness was not considered and that they were unaware of any bodies/committees representing them. The continuous sourcing of feedback from patients regarding the facility, resources, processes and procedures and the formulation of patient committees could change the status quo from exclusion to inclusion and improve patients’ experiences. Another strategy to assist new patients and address the staff shortages at the ARV clinics could be the recruitment of ‘patient advocates’, ‘patient ambassadors’, ‘patient representatives’, ‘expert patients’, ‘mentor patients’ who have several years’ experience on ART. Such strategies have been positively recorded in other studies [61, 62].

In summary, this study’s findings highlighted varying systems and processes at the ARV clinics, although all four sites provide free ART and are based within KZN Department of Health (DOH) hospitals. Such variations could affect patients’ experiences, contribute to lost files, perpetuate a stigmatised environment and patient dissatisfaction.

## Experiences of the ART policy and its implementation

The South African government’s decision to rollout out a public ART programme was well received and has succeeded in many PLHIV enrolling on the programme with much of the health budget being allocated to the programme. Though the initial policy and its subsequent revisions aimed to embrace all PLHIV and improve patients’ quality of life, all patients did not experience the policy in the light of the positive outcomes it was meant to achieve. A reason could be that the policy was not adequately communicated to patients as the results reflect patients reporting being submissive in a paternalistic programme.

## Patients’ experiences

The quantitative results provided an overview of patients’ experiences, whilst the qualitative results provided a rich in-depth understanding of both positive and negative experiences. The positive areas were that routine blood results were mostly available, most staff greeted patients, there were sufficient nursing staff, patients were satisfied with the time spent with doctors, clean clinics and counselling areas were private and safe, patients appreciated being allowed to ask questions and receiving free ART.

Areas of concern which the study results highlight are poor relationships with nurses rather than doctors; negative staff attitudes; disrespectful staff; information gaps; inadequate information shared by counsellors at times; varying appointments, data capture and registration systems; varying ARV collection frequencies, routine health tests and processes per site; absence of patient committees and patient representatives as well as inflexible systems. Though the Department of Health’s protocol guides all four ARV clinics, the execution of same differs per site. The study results position the programme as a one size fits all rather than a programme that caters to patients' uniqueness and humanness. It does not promote patient engagement and relationship building which contradicts patient-centred care. Such results negate the importance of patients’ views on their care and treatment, even though previous studies have highlighted the link between poor provider-patient relationships, satisfaction, retention, and adherence [63, 64].

In summary, this study emphasizes patients’ appreciation for free lifesaving ARV drugs and mostly reports negative patients’ experiences.

## Limitations of the study

A larger sample including patients under 18 years and the inclusion of more ARV clinics could have yielded more results. In addition, the PI and research assistants’ disclosure of their HIV-positive status could have posed a bias whilst also encouraging reliable and valid responses.

## Recommendations

Future studies could include staff’s experiences of the public ART programme. As this study includes four sites, each with unique processes and systems with all 400 respondents completing the same questionnaire, it could be adapted to suit patients’ experiences of the ART programme at other sites. Nurse initiated management of antiretroviral therapy (NIMART) trained nurses could assist with the doctors’ shortfall. Patients should be included in workshops and training sessions to ensure the gatekeepers understand their ART experiences and the impact thereof.

## Conclusion

Although the public ART programme is in existence for over a decade, many respondents did not describe positive experiences. Hence, rendering patient-centred care mere rhetoric rather than reality. Patients articulated their successful and challenging experiences in an endeavour to be heard. The results reflect opportunities for health systems’ strengthening at some of the ARV clinics. They further create an opportunity for best practices between sites to be shared (cross-learning) and training to be implemented to directly improve patients’ experiences, service delivery and influence adherence and retention which are relevant to patient-centred care.

## Data Availability

All generated and available data for this paper has been included.
